# Pyrosequencing Reveals Changes in Soil Bacterial Communities after Conversion of Yungas Forests to Agriculture

**DOI:** 10.1371/journal.pone.0119426

**Published:** 2015-03-20

**Authors:** Marcela S. Montecchia, Micaela Tosi, Marcelo A. Soria, Jimena A. Vogrig, Oksana Sydorenko, Olga S. Correa

**Affiliations:** Instituto de Investigaciones en Biociencias Agrícolas y Ambientales (INBA-CONICET/UBA), Cátedra de Microbiología Agrícola, Facultad de Agronomía, Universidad de Buenos Aires, Ciudad Autónoma de Buenos Aires, Argentina; University of Tartu, ESTONIA

## Abstract

The Southern Andean Yungas in Northwest Argentina constitute one of the main biodiversity hotspots in the world. Considerable changes in land use have taken place in this ecoregion, predominantly related to forest conversion to croplands, inducing losses in above-ground biodiversity and with potential impact on soil microbial communities. In this study, we used high-throughput pyrosequencing of the 16S ribosomal RNA gene to assess whether land-use change and time under agriculture affect the composition and diversity of soil bacterial communities. We selected two areas dedicated to sugarcane and soybean production, comprising both short- and long-term agricultural sites, and used the adjacent native forest soils as a reference. Land-use change altered the composition of bacterial communities, with differences between productive areas despite the similarities between both forests. At the phylum level, only Verrucomicrobia and Firmicutes changed in abundance after deforestation for sugarcane and soybean cropping, respectively. In cultivated soils, Verrucomicrobia decreased sharply (~80%), while Firmicutes were more abundant. Despite the fact that local diversity was increased in sugarcane systems and was not altered by soybean cropping, phylogenetic beta diversity declined along both chronosequences, evidencing a homogenization of soil bacterial communities over time. In spite of the detected alteration in composition and diversity, we found a core microbiome resistant to the disturbances caused by the conversion of forests to cultivated lands and few or none exclusive OTUs for each land-use type. The overall changes in the relative abundance of copiotrophic and oligotrophic taxa may have an impact in soil ecosystem functionality. However, communities with many taxa in common may also share many functional attributes, allowing to maintain at least some soil ecosystem services after forest conversion to croplands.

## Introduction

The Andean tropical and subtropical rainforests of South America extend along the eastern slope of Andes, from Venezuela and Colombia to its southern limit in Northwest Argentina (NWA), where they are known as Southern Andean Yungas [1–3]. The latter is an ecoregion that covers less than 2% of the Argentinean territory but constitutes one of its major hotspots of biodiversity [4]. Over the last decades, considerable changes in land use have taken place in the subtropical region of NWA, which carried an increased rate of deforestation and agricultural intensification that affected Yungas and Chaco forests particularly [5–7]. This type of changes in land use causes a noticeable loss of plant and animal biodiversity but it is still unclear if the impact to above-ground diversity is accompanied by changes in soil microbial diversity. In fact, very little is known regarding below-ground biodiversity in the Yungas ecoregion, which has been approached only in a few studies focused in fungi [8, 9]. Soil microbial diversity has been underestimated in this region despite the fact that microorganisms, and especially bacteria, are the most abundant and diverse organisms in terrestrial ecosystems and are intimately involved in ecosystems functioning [10]. As a matter of fact, ecosystem functional changes after the conversion of subtropical forests to croplands have already been reported [11].

It has been proved that conversion of tropical forest lands to agriculture alters the structure of soil bacterial communities, but those changes did not necessarily imply a loss of bacterial diversity. In fact, some studies have reported no differences or even higher local diversity of bacterial communities in agricultural soils when compared to adjacent natural forest soils [12–16]. Nevertheless, a recent study of soil bacterial diversity in Amazon rainforests converted to farmland reports that even though local taxonomic and phylogenetic diversity of soil bacteria increases after conversion, the homogenization of bacterial communities results in a net loss of microbial diversity [17]. In these soils, forest-to-pasture conversion also alters microbial functional diversity of genes linked to carbon and nitrogen cycling [18].

The impact of land-use change on microbial diversity should receive special attention in tropical and subtropical regions, where land deforestation for agriculture still occurs at high rates [19]. Under this climate region, the resulting agroecosystems are particularly susceptible to soil degradation, mainly due to the loss of nutrients and organic matter that result from higher mineralization rates and outputs exceeding inputs. It has been largely reported that conversion of primary forest into cropland induces decreases in total organic carbon stocks and sometimes total nitrogen, and that those losses are bigger and more consistent than in a conversion from forest to uncultivated grazing land [20, 21]. These results were also found in tropical Andean rainforests from Ecuador, where annual cropping resulted more detrimental to organic carbon and nutrient stocks than perennial crops or pastures [22].

Despite the fact that the diversity-functioning relationship remains controversial and prompts many discussions [23], it is still accepted that a diverse microbial community is more resistant and resilient to stressing situations, and therefore more capable of sustaining soil functions [24, 25]. Thus, as one of the major goals in soil conservation is maintaining ecosystem functioning, one of the foundations for sustainable agriculture should be to preserve microbial diversity [26].

In a previous study carried out in the Argentinean Yungas, we used traditional microbial community profiling methods (denaturing gradient gel electrophoresis of 16S rRNA genes, community-level physiological profiling and phospholipid fatty acid analysis) to show structural and functional differences between microbial communities from agricultural and pristine soils [27]. To assess more extensively the molecular diversity and taxonomy of soil microbial communities, a powerful tool is the pyrosequencing of the 16S ribosomal RNA gene, which has become one of the most popular culture-independent methods [28]. Although several studies have used high-throughput sequencing of soil bacterial communities to uncovering the effects of agricultural land use in rainforests, few have studied paired sites where pristine forest soils have been converted directly to agricultural soils [15–17, 29]. Furthermore, to date, there are no such studies carried out in the Argentinean Yungas. In this context, we focused on the response of diversity and composition of soil bacterial communities, assessed by 454 pyrosequencing, to the conversion of forest to farmlands. Our study was carried out in two areas of the Yungas ecoregion currently dedicated to sugarcane and soybean production, both under short- and long-term agriculture, using the adjacent native forest soils as a reference. According to our previous results, we hypothesize that land-use change will alter the composition of bacterial communities, with lasting negative effects on diversity relative to primary forest soils.

## Materials and Methods

### Site description

According to Olson et al [3], the Argentinean Yungas are grouped within the tropical and subtropical moist broadleaf forests biome. It extends between 400 and 3000 meters above sea level (m a.s.l.) and it is located on the southernmost limit of the Neotropical Cloud Forests, a major system in Latin America [30]. The ecoregion of Yungas is among the places sheltering the highest biodiversity of the country. In Argentina, it takes place in the Northwest (NWA), along a narrow and discontinuous belt throughout the provinces of Salta, Jujuy, and Tucumán, from 22° to 28° 15′ S. The Yungas has been ecologically subdivided into northern, central, and southern sectors, according to the orographic pattern of two mountain ranges called Sierras Subandinas and Sierras Pampeanas, which precede the Andes Mountains to the West [30]. Rainfall is markedly seasonal (dry winter and wet summer) and varies from 700 to 2000 mm per year. Mean annual temperature ranges from 18 to 20°C.

The pedemontane forest of the Yungas, the lowest altitudinal vegetation-level, is the most threatened by changes in land use, being agriculture one of the main activities, with sugarcane and soybean among the most relevant cropping systems. It occupies the lower slopes of the Subandine Sierras and Sierra of Calilegua and part of the plain in transition to the Semi-arid Chaco. The altitude of the pedemontane forest ranges from 700 m a.s.l. in the West to 400 m a.s.l. in the East, following a W-E gradient of annual rainfall from 1000 to 700 mm, respectively. A more detailed description of the studied area can be found elsewhere in the literature [31, 32].

### Sample collection

In May 2011, at the end of the rainy season, agricultural and forest soils samples were collected from various sites across the central-northern region of the Argentinean Southern Andean Yungas, covering two areas in Jujuy and Salta provinces, dedicated to sugarcane and soybean crops, respectively. Agricultural sites were formerly pedemontane forest lands of the Yungas deforested in different moments of a 30 and 100-year period for soybean and sugarcane cultivated areas, respectively. Primary forest remains adjacent to agricultural plots. None of the sampling areas belonged to protected areas and we obtained permissions from staff responsible for the different agricultural farms. For each area, we selected three sets of paired sites, each set including at least one site for each of the three land-use categories: long-term agriculture (30–100 years since clearing), short-term agriculture (2–5 years since clearing) and undisturbed pristine forest (Table 1).

**Table 1:** Description of soil samples according to province, farm and land use.

Sample ID^a^	SRA code^b^	Land use and management history	Coordinates and elevation (m a.s.l.)
J1-F	HM	Pristine pedemontane forest	24°03′55.3"S 64°38′43.0"W, 391 m
J1-STA	H2	5 years of sugarcane monoculture	24°04′22.2"S 64°39′30.2"W, 390 m
J1-LTA	H3	30 years of sugarcane monoculture	24°04′39.1"S 64°39′39.2"W, 390 m
J2-F	n/a	Pristine pedemontane forest	23°31′29.8"S 64°15′05.6"W, 391 m
J2-STA	T2	5 years of sugarcane monoculture	23°31′40.0"S 64°16′06.5"W, 391 m
J2-LTA	T1	40 years of sugarcane monoculture	23°31′42.1"S 64°16′12.3"W, 380 m
J3-F	PM	Pristine pedemontane forest	23°51′14.3"S 64°39′20.2"W, 391 m
J3-STA	P2	2 years of sugarcane monoculture	23°51′01.4"S 64°39′05.4"W, 370 m
J3-LTA	P1	40 years of sugarcane monoculture	23°50′33.4"S 64°39′25.3"W, 376 m
J3-LTA2	SC1009	100 years of sugarcane monoculture	23°50′03.4"S 64°46′45.6"W, 370 m
J3-LTA07^c^	SC1007	100 years of sugarcane monoculture	23°50′03.4"S 64°46′45.6"W, 370 m
S1-F	ISM	Pristine pedemontane forest	24°52′26.4"S 64°12′10.6"W, 441 m
S1-STA	122	3 years of soybean monoculture	24°51′49.0"S 64°14′51.7"W, 487 m
S1-LTA	131	30 years of agricultural use: 26 years of soybean monoculture (1985–2011)	24°52′30.5"S 64°11′38.0"W, 514 m
S2-F	103M	Pristine pedemontane forest	24°54′01.9"S 64°20′13.5"W, 577 m
S2-STA	103	5 years of soybean monoculture	24°54′08.9"S 64°19′55.2"W, 578 m
S2-LTA	20	30 years of agricultural use: 23 years of soybean monoculture (1987–2010) and maize during the last year	24°53′11.9"S 64°12′15.4"W, 458 m
S3-F	PC4M	Pristine pedemontane forest	24°51′59.4"S 64°19′09.9"W, 540 m
S3-STA	PC4	5 years of soybean monoculture	24°51′39.8"S 64°19′02.6"W, 503 m
S3-LTA	107B	30 years of agricultural use: 23 years of soybean monoculture (1986–2009) and soybean/maize during the last 2 years	24°52′34.5"S 64°19′00.2"W, 538 m

^a^Soil sample designation refers to their geographical origin (J: Jujuy, S: Salta), farm identification (1 to 3) and land use (F: forest, STA: short-term agriculture, LTA: long-term agriculture).

^b^Sample code in Sequence Read Archive—NCBI.

^c^Sampled in March 2007.

n/a: non-available. Missing data in pyrosequencing analysis.

By choosing sets of paired sites, we were able to control, at least partially, soil heterogeneity. This is particularly relevant in observational studies like ours, since many variables cannot be manipulated. At each site, a squared sampling plot of 1 ha was selected and, within each plot, five sampling points were established (four in each of the corners and one in the center). In each of the sampling points, we took composite samples consisting of 16 soil cores (10 cm depth, 5 cm diameter). Samples were taken from the inter-row zone, previously removing litter. Similarly, zones intimately linked with roots were avoided in forest sites. For this study, we focused on intersite variability and not in the variability of bacterial communities within plots, thus, equal amounts of the five composite samples were combined in a single composite sample per sampling point. With this method, used in a similar study [15], we intended to assess how land-use itself influences the structure and diversity of bacterial communities. Each sample was then thoroughly homogenized and divided in two parts; one was sent to a commercial laboratory (Laboratory of Soil and Water, INTA Salta) to be processed for chemical analyses using standard procedures [33], whereas the other was kept field-moist, sieved through a 2 mm mesh and stored at -80°C until DNA extraction. The main chemical properties of soils are summarized in S1 Table.

### DNA extraction, PCR amplification and barcoded pyrosequencing of the bacterial 16S rRNA gene

Total DNA was extracted from 0.25 g of each soil sample using the Power Soil DNA isolation kit (Mo Bio Laboratories, Inc.) according to manufacturer’s instructions. The concentration and quality (A_260_/A_280_) of the extracted DNA were determined using a GeneQuant RNA/DNA calculator spectrophotometer (Pharmacia Biotech). Amplicons for barcoded pyrosequencing were generated by PCR using the primer set 515F (GTGCCAGCMGCCGCGGTAA) and 806R (GGACTACVSGGGTATCTAAT), targeting the V4 region of the bacterial 16S rRNA gene. Unidirectional sequencing of the amplicon libraries was performed from the forward primer at Macrogen Inc. (Seoul, South Korea) using the 454-GS-FLX Titanium system. Sequences were deposited with the NCBI under BioProject accession number PRJNA260275.

### Processing of pyrosequencing data

All sequence processing was done using mothur version 1.31.2 [34]. Sequences were discarded if they contained more than two differences to the primer sequence or more than one difference to the barcode. The resulting sequences were denoised with the PyroNoise method as implemented by mothur. In addition sequences with more than eight homopolymers or a length of less than 200 nucleotides were discarded. Sequences that passed these quality filters were aligned using the Silva project (http://arb-silva.de/) alignment database as a reference. Chimeras were removed using the UCHIME method implemented in mothur and using the Silva database as a reference. For the 19 libraries included in this study, the number of raw reads varied between 20639 and 3712. After quality filtering, libraries for each site were normalized to 3622 sequences. Pairwise distances matrixes were built and the sequences were clustered into OTUs at 95% similarity. OTUs that had summed nine or less reads across all the samples in an area were considered rare and removed for the analyses unless specified.

### Data analyses

For phylogenetic identification, sequences and OTUs were classified with the Wang method implemented in mothur and the database provided by the Ribosomal Database Project (RDP; http://rdp.cme.msu.edu). Using mothur we obtained values for the Shannon [35] and Inverse Simpson [36] alpha diversity indices, as well as the Chao1 [37] and ACE [38] measures of OTU richness and dissimilarity matrices (Bray-Curtis and weighted UniFrac). To explore whether bacterial community composition clustered according to land use we plotted the results of non-metric multidimensional scaling (NMDS) and principal coordinates analyses (PCoA) using the Bray-Curtis and weighted UniFrac dissimilarities. These results were further evaluated with AMOVA (analysis of molecular variance) and permutation tests on the UniFrac calculations. Relationship between community composition (Bray-Curtis and unweighted UniFrac measures) and environmental factors was analyzed using constrained analysis of principal coordinates (CAP) [39]. To identify the OTUs that were more influential in the ordinations we carried out an analysis of indicator species using the method described by Dufrene and Legendre (1997) [40] with the *indval* function implemented in the R package *labdsv* [41]. The distribution data for the OTUs were first ANOVA-filtered to reduce the number of comparisons. Then, these candidate OTUs were analyzed with the *indval* function and the list of *p*-values obtained was corrected to remove false positives originating from the multiple comparisons with a “false discovery rate” correction. To test changes in distribution of phyla across land uses, we used generalized models for data with negative binomial distributions to account for overdispersion [42]. The models were built and tested with the *glm*.*nb* function implemented in the *MASS* package of R (version 3.1.1, R Core Team, 2014) [43].

Finally, the analysis of chemical data to compare the two areas was carried out both with a principal components ordination and with additional paired t-tests to check for significant differences. For each area, differences among land uses for each chemical variable were analyzed using Analysis of Variance (ANOVA).

## Results

### Soil chemical properties

**Fig 1:**
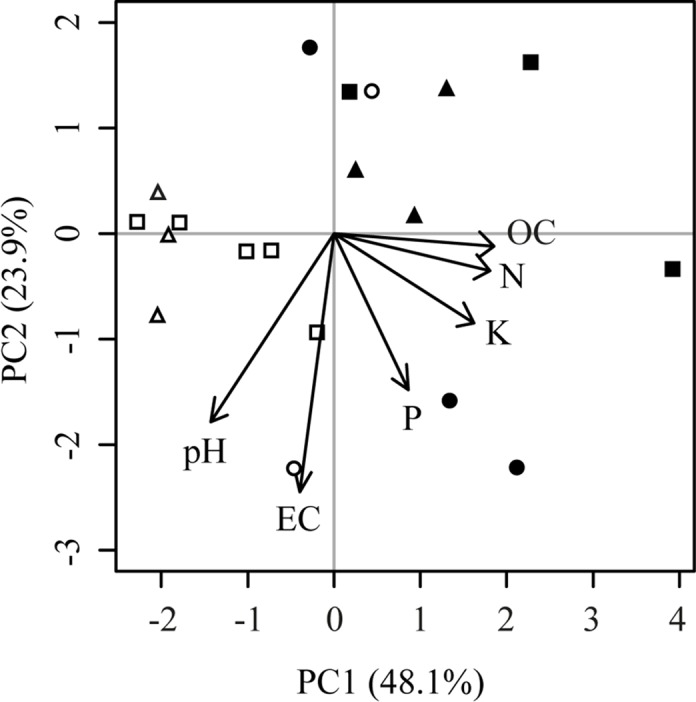
Principal component analysis of soil chemical variables. Empty and filled symbols correspond to soils from Jujuy and Salta, respectively. Shape of symbol represents land use: forests (circles), short-term (triangles) and long-term agriculture (squares).

A principal components ordination was built using chemical data of soils (Fig. 1). Irrespectively of land use, soils were mainly separated into the two different areas of Jujuy and Salta. This ordination responded to differences in soil total nitrogen (N) and organic carbon (OC), both higher in soils from Salta. A *t-*test revealed lower pH (*P* = 0.006) and higher extractable potassium (K) (*P*<0.001) in soils from Salta.

When analyzing soil chemical properties individually for each area, we found a significant decrease in total N and OC in response to agricultural activity in both Salta (*P*-values 0.0021 and 0.0114, respectively) and Jujuy (0.0002 and 0.0006, respectively). We found no significant effect of land use in pH and other chemical properties (*P*>0.05). Despite the fact that there was not a particular response of pH to land use, we did observe that pH values tended to be similar between long-term agriculture soils from each area (S1 Table).

The differences in soil chemical properties between the two areas, and the fact that they are dedicated to different cropping systems, encouraged us to carry out separate analyses of microbial communities: Salta, an area destined to annual crops with soybean as the main summer crop, and Jujuy, fully-destined to sugarcane sowing.

### Composition of soil bacterial communities

In both Salta and Jujuy data sets, approximately 20% of the reads were assigned to rare OTUs with very few reads, and the rank species distribution plots had very long tails. Although these are expected features in soil samples, low count OTUs are still very susceptible to sampling errors and, in consequence, they can adversely affect the statistical analysis. Therefore, we decided to remove all OTUs with nine or less reads before further analysis, assuming the trade-off that real differences could be overlooked.

**Fig 2:**
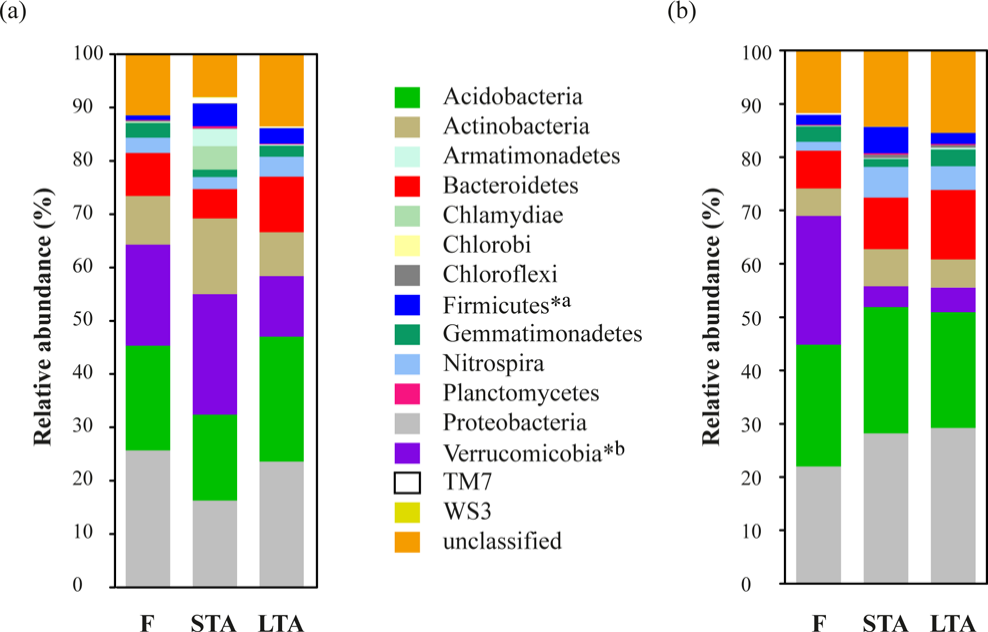
Composition of soil bacterial communities at the phylum level in both areas: Salta (a) and Jujuy (b). Average relative read abundance of bacterial phyla in forest and agricultural soils. *^a^ and *^b^ indicate significant differences between land uses in Salta and Jujuy, respectively. F: forest, STA: short-term agriculture, LTA: long-term agriculture.

Composition at the phylum level of the whole soil bacterial communities are shown in Fig. 2.

A total of 15 phyla were found within the soil samples from Salta. At the phylum level, irrespectively of land use, communities were dominated by Proteobacteria (21.8%), Acidobacteria (19.8%), Verrucomicrobia (17.6%), Actinobacteria (10.6%) and Bacteroidetes (8.0%). A fraction of reads (11.0%) remained as unclassified. In the productive area of Jujuy, a total of 14 phyla were found within the classified sequences. At the phylum level, communities showed a similar although not identical profile to that of Salta. Communities in Jujuy were dominated by Proteobacteria, which on average had a higher frequency (26.4.8%) than in Salta. The next most frequent phylum was Acidobacteria (22.8%), followed by Verrucomicrobia (10.9%), Bacteroidetes (9.9%) and Actinobacteria (5.8%), the latter being less frequent than in Salta, as Verrucomicrobia. A similar fraction of reads (13.8%) remained as unclassified.

When the effect of land use on relative abundance of phyla was evaluated, only Firmicutes and Verrucomicrobia showed changes in their abundances according to land use among the most abundant phyla (greater than 2%). In Salta soils, Firmicutes were less frequent in forest sites (*P* = 0.02), while Verrucomicrobia were more frequent in forest soils from Jujuy (*P* = 0.003).

### OTUs distributions by land use

Although most sequences were classified at phylum and class levels, many of them could not be classified at genus level. Therefore, we conducted a taxon-independent analysis using OTUs as the unit of analysis for the in-depth ecological analysis of bacterial communities.

Once rare units were removed (OTUs with nine or less reads), most of the OTUs showed representatives in every land-use type in both areas (see Venn diagrams in S1 Fig.). However, in Jujuy area some groups of OTUs were restricted to agricultural soils (S1b Fig.). Moreover, the proportion of OTUs exclusive to agricultural soils was much higher in this area than in Salta (105/504 vs. 12/415, respectively). In Salta, there were no OTUs exclusive to a single land use (S1a Fig.).

### Richness and diversity estimates

In order to inquire into the apparent uniformity suggested by the Venn diagrams, we calculated alpha and beta diversity in several ways to search for differences between bacterial communities from contrasting land uses (Table 2).

**Table 2:** Richness estimates and diversity indices for soil samples under different land uses from Salta and Jujuy.

Sample ID^a^	Richness			Diversity	
	Observed	Chao	ACE	Shannon	Inverse Simpson
S1-F	304	346 [327–382]	352 [334–381]	4.5 [4.42–4.55]	21.0 [18.79–23.74]
S1-STA	342	382 [364–416]	385 [369–412]	5.1 [5.04–5.13]	89.8 [83.14–97.55]
S1-LTA	338	384 [364–420]	391 [372–421]	4.8 [4.71–4.82]	43.8 [39.88–48.56]
S2-F	338	376 [359–408]	387 [369–415]	4.5 [4.43–4.57]	17.2 [15.40–19.38]
S2-STA	333	370 [353–402]	376 [360–403]	4.5 [4.45–4.59]	18.3 [16.40–20.64]
S2-LTA	322	376 [353–418]	387 [364–423]	4.6 [4.57–4.69]	36.2 [33.08–39.99]
S3-F	333	361 [348–387]	369 [355–392]	4.8 [4.79–4.90]	51.6 [47.08–57.14]
S3-STA	348	402 [379–442]	402 [383–433]	4.9 [4.82–4.92]	55.9 [51.49–61.11]
S3-LTA	342	374 [359–403]	380 [366–405]	4.9 [4.88–4.98]	57.2 [51.84–63.75]
J1-F	209	243 [225–279]	241 [227–266]	3.7 [3.62–3.77]	9.0 [8.24–9.91]
J1-STA	396	441 [422–474]	454 [434–484]	5.1 [5.01–5.11]	70.0 [64.28–76.87]
J1-LTA	408	475 [448–520]	475 [453–509]	5.1 [5.08 5.18]	77.6 [71.56–84.67]
J2-STA	374	442 [415–489]	438 [416–472]	5.0 [4.90–5.01]	58.1 [53.01–64.28]
J2-LTA	385	427 [409–458]	438 [419–466]	5.0 [4.97–5.07]	69.0 [63.60–75.33]
J3-F	361	412 [391–449]	423 [401–455]	4.7 [4.65–4.77]	40.1 [36.87–44.00]
J3-STA	372	427 [404–469]	425 [406–455]	5.0 [4.95–5.06]	55.4 [50.15–61.97]
J3-LTA	391	443 [421–481]	443 [425–472]	5.2 [5.14–5.24]	83.3 [75.94–92.32]
J3-LTA2	387	459 [430–507]	458 [434–493]	5.0 [4.95–5.06]	65.8 [60.68–71.90]
J3-LTA07	397	465 [437–511]	461 [440–494]	5.1 [5.03–5.13]	74.3 [68.94–80.61]

Values in brackets are the lower and upper limits of 95% confidence intervals.

^a^Soil sample designation refers to their geographical origin (J: Jujuy, S: Salta), farm identification (1 to 3) and land use (F: forest, STA: short-term agriculture, LTA: long-term agriculture).

In Salta, the analyses of several richness and diversity measures suggested a slight increase in richness in communities from short-term agriculture soils, and lower community diversity in forest soils, although these were tendencies and not statistically significant results (Table 2). In addition, the rarefaction curves suggested that not all the expected richness of the bacterial communities is accounted in the sequenced samples (S2a Fig.). On the other hand, in Jujuy the lowest bacterial richness and diversity belonged to one of the forest soils (Table 2). As in Salta soils, all rarefaction curves based on observed richness were unsaturated (S2b Fig.), indicating that the surveying effort could not cover all the taxonomic diversity inhabiting those soils. Considering the chronosequence as a whole, we observed an increase in diversity and, to a lesser extent, richness. Nonetheless, we must be cautious because we were able to analyze only two forest sites in this area.

**Fig 3:**
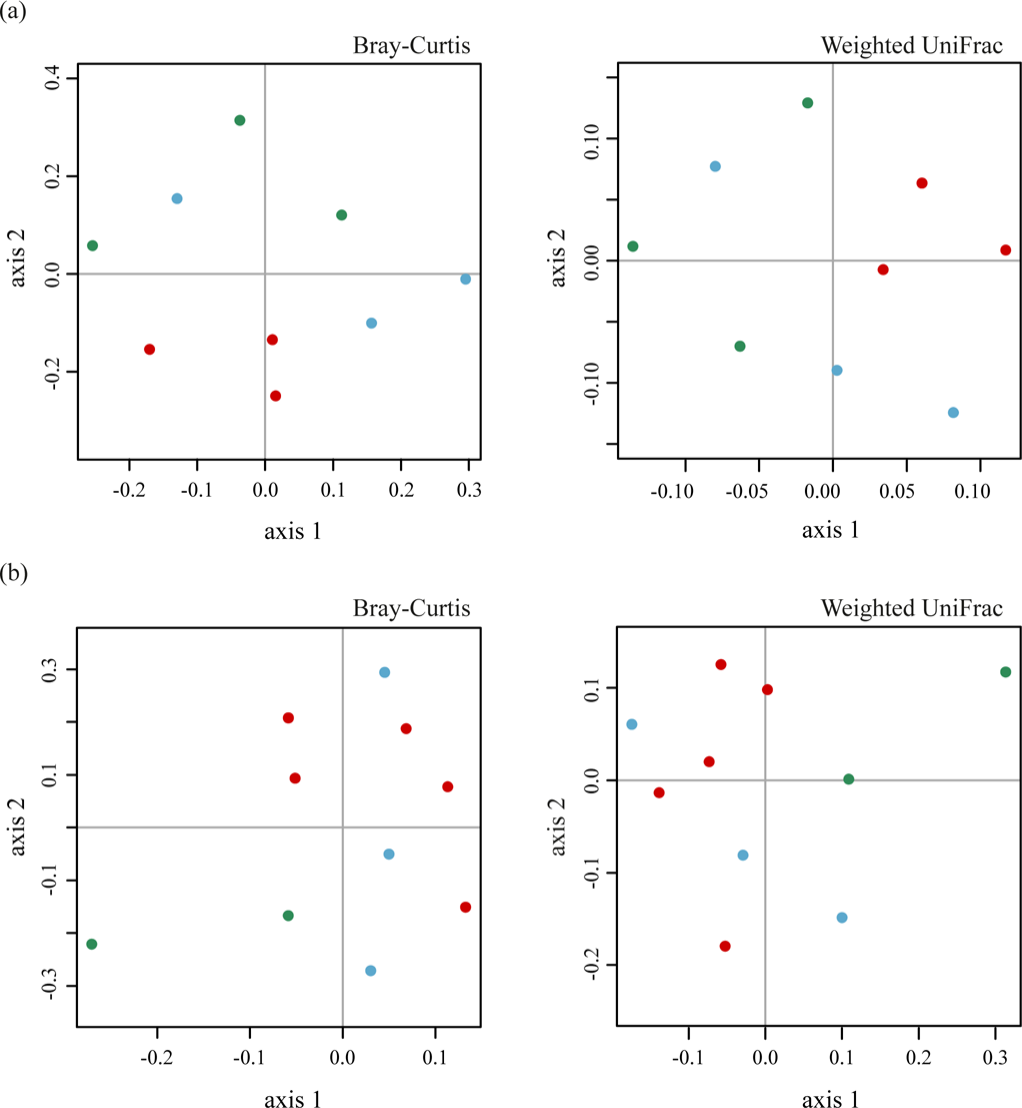
Nonmetric multidimensional scaling (NMDS) plots derived from pairwise Bray-Curtis and weighted UniFrac distances between bacterial communities from forest and agricultural soils from Salta (a) and Jujuy (b). Symbols are coded by land use (green: forest, blue: short-term agriculture, red: long-term agriculture).

To visualize overall similarities and differences in community structure between soil samples, pairwise Bray-Curtis and weighted UniFrac dissimilarities were calculated and ordinated in two-dimensional NMDS plots (Fig. 3).

The NMDS plot ordinations of soils from Salta area derived from both metrics, taxonomic (Bray-Curtis distances) and phylogenetic (weighted UniFrac), showed similar patterns (Fig. 3a). Thus, bacterial communities from long-term agriculture soils were similar and clustered together, while for the other uses the distribution is more widespread and shows no aggregation pattern (R^2^ = 0.76; Bray-Curtis and 0.75; weighted UniFrac).

The previous ordinations suggested that the structure of soil bacterial communities from Salta differed between sites with different land use. However, we must recall that the Venn diagram had suggested that many members of different OTUs were present in all land-use types (S1 Fig.). Thus, in order to test the hypothesis that despite the ubiquity of OTUs, there could still be quantitative differences in their distribution, we performed two permutation-based statistical tests: UniFrac significance test and analysis of molecular variance (AMOVA). The Unifrac test revealed differences between land uses (*P*<0.005). Pairwise scores showed a slightly larger difference between bacterial communities from the forest (F) and long-term agriculture (LTA) than between forest and short-term agriculture (STA) (UniFrac values: STA-LTA = 0.0967, STA-F = 0.1036, LTA-F = 0.1267). The overall AMOVA test also showed a highly significant effect of land use (*P*<0.005). After applying the Bonferroni correction for multiple tests, individual contrasts for pairwise comparisons were also highly significant (*P*<0.005).

In Jujuy, NMDS plots using Bray-Curtis and weighted Unifrac indices (Fig. 3b showed that bacterial communities of forest soils were separated from those of agricultural sites, which in turn were close to each other (R^2^ = 0.857; Bray-Curtis and 0.816; weighted UniFrac). Likewise, the variation in bacterial community composition was higher in forest than in agricultural soils. The Venn diagram also showed a similar pattern between cultivated soils, since a large proportion of OTUs was present in both short- and long-term sites (S1b Fig.). The Unifrac test revealed a significant effect of land use (*P*<0.005), and the pairwise scores showed that the largest differences were between forest and the two different agricultural sites (UniFrac values: STA-LTA = 0.1221, STA-F = 0.2599, LTA-F = 0.2555). The same significant effect of land use was observed with the AMOVA test (*P*<0.005) and then individually with the Bonferroni-corrected pairwise contrast (*P*<0.005).

Summarizing, the analysis of beta diversity showed similar results in both areas: the composition of bacterial communities from cultivated soils was more similar than in native forest soils. This result reflects a decrease in beta diversity after land-use change.

### Community structure and soil properties

To determine whether the patterns observed in the ordinations with the Bray-Curtis and unweighted UniFrac measures could be related to soil chemical parameters or land use, we performed a constrained analysis of principal coordinates (CAP). Since the organic carbon and total nitrogen contents were highly correlated with changes in land use, we excluded the former variables from the analyses and used instead the land use as a single categorical variable. Also, as P and K contents were significantly correlated, only P was considered. The structure of soil bacterial communities from Salta did not show any association with any of the factors considered, the *P*-value of the ANOVA tests of the CAP model with the Bray-Curtis dissimilarity was 0.275 and with the weighted UniFrac dissimilarity was 0.411. In the same direction, a stepwise procedure of variable elimination and addition indicated that none of the factors considered influenced the ordinations. In contrast, the beta diversity of the soil bacterial communities from Jujuy was influenced by soil pH and land use (*P* = 0.011) when the Bray-Curtis measure was considered, and only by pH with the weighted UniFrac measure (*P* = 0.03).

### Indicator taxa of changes in land use and soil pH

We carried out an IndVal analysis to OTU distribution data in order to search for indicators of changes in land use. Based on the results found in the CAP analysis from Jujuy, we looked for indicator OTUs of soil pH as well. Only a few significant indicators of changes in land use or soil pH were identified in each area (Table 3).

**Table 3:** Results of Indicator Species Analysis of changes in land use or pH in Jujuy and Salta.

Taxon	Size^a^	IndVal^b^	*P* (corrected)	Higher in
**Jujuy**				
Spartobacteria	1261	0.8525	0.035	Forest soils
Actinobacteria	39	0.6716	0.035	Forest soils
Alphaproteobacteria	53	0.5650	0.032	Short-term agricultural soils
Acidobacteria Gp3	46	0.5801	0.016	Short-term agricultural soils
Gammaproteobacteria	40	0.5594	0.035	Short-term agricultural soils
Alphaproteobacteria	23	0.4989	0.035	Short-term agricultural soils
Betaproteobacteria	180	0.5049	0.035	Long-term agricultural soils
Gemmatimonadetes	64	0.7115	0.016	Long-term agricultural soils
Acidobacteria Gp10	39	0.6108	0.032	Long-term agricultural soils
Deltaproteobacteria	33	0.6247	0.016	Long-term agricultural soils
Acidobacteria Gp7	173	0.6168	0.036	Soils with higher pH
Gammaproteobacteria	135	0.5886	0.036	Soils with higher pH
Acidobacteria Gp6	131	0.5976	0.036	Soils with higher pH
**Salta**				
*Chitinophagaceae*	16	0.696	0.048	Forest soils
Betaproteobacteria	23	0.677	0.048	Short-term agricultural soils
Betaproteobacteria	24	0.649	0.048	Short-term agricultural soils
Nitrospira	52	0.667	0.048	Long-term agricultural soils
Myxococcales	36	0.500	0.048	Long-term agricultural soils
Actinobacteria	31	0.525	0.048	Long-term agricultural soils
Acidobacteria Gp4	67	0.513	0.036	Soils with higher pH

Only significant OTU indicators containing 20 or more sequences and identified at phylum level and below are shown.

^a^Total number of reads corresponding to the OTU that represents the specific group of soil samples.

^b^Indicator value index.

In Jujuy, two indicator taxa were identified for forest soils and four indicator taxa for each of the agricultural soils. The forest soils were dominated by OTUs belonging to Spartobacteria and Actinobacteria. In both agricultural sites we found indicator OTUs from Proteobacteria and Acidobacteria, but belonging to different classes or subgroups, respectively. While OTUs from Alphaproteobacteria, Gammaproteobacteria and Acidobacteria Gp3 were indicators of short-term agricultural sites, long-term agricultural sites were associated with OTUs from Betaproteobacteria, Deltaproteobacteria and Acidobacteria Gp10. Besides the members from those two phyla, long-term sites were also characterized by an OTU belonging to Gemmatimonadetes. Regarding indicators of changes in soil pH, we found three OTUs related to soils with higher pH values, belonging to Acidobacteria Gp6, Acidobacteria Gp7 and Gammaproteobacteria.

In Salta area, we found less indicator OTUs for all the surveyed soils and, in general terms, corrected *P*-values were higher than in Jujuy IndVal analysis (Table 3). Unlike what we observed in Jujuy, there was a sole indicator OTU of forest soils, identified as a member of the family *Chitinophagaceae*. Short-term agricultural soils were characterized by two OTUs belonging to Betaproteobacteria, while long-term sites were associated with three OTUs belonging to Nitrospira, Actinobacteria and Myxococcales. Only one OTU, belonging to Acidobacteria Gp4, was related to soil pH in Salta, being indicative of higher pH values.

## Discussion

In this study, we reported the response of soil bacterial communities to change in land use and time under agriculture (short- and long-term) in productive fields from Jujuy (sugarcane) and Salta (soybean) in the Argentinean Yungas. Conversion of forest to cropland altered the composition and diversity of soil bacterial communities. However, we detected a core microbiome resistant to disturbances caused by deforestation and agriculture. Alpha and beta diversity were affected differentially, and there were also differences between productive areas. Alpha diversity showed no response to land use in Salta, but it was higher in agricultural soils from Jujuy. However, phylogenetic beta diversity, measured as variation, was reduced in both productive areas. The latter result implies a homogenization of soil bacterial communities in response to cultivation and a net loss of diversity. Additionally, the association between the structure of bacterial communities and soil parameters differed between Salta and Jujuy. In Salta, changes in microbial community structure could not be attributed to land-use dependent soil parameters, while in Jujuy it was related not only to land use but also to soil pH. Indicator species analysis showed that few OTUs could function as an indicator of changes in land use in Jujuy, while in Salta results should be carefully considered because no association was found between the structure of soil bacterial communities and soil properties.

High-throughput sequencing of 16S rRNA gene V4 region showed that, although forest soils from Jujuy and Salta areas were dominated by the same bacterial phyla, agricultural use impacted differently on the relative abundance of different taxa in each one. In Salta, an area destined to soybean crop, the only difference we found at phylum level was for Firmicutes, which were more abundant in agricultural soils. A similar result at whole-phylum level was found by Rodrigues et al. [17] in Amazon soils, where Firmicutes showed the largest proportional increase after land-use conversion (forest to pasture). These shared tendencies can be explained by the fact that members of this phylum might have advantages in both pasture and agricultural systems, where the soil surface temperatures can vary sharply through the day, due to their remarkable resistance to desiccation. Despite this coincidence, these authors also found that Acidobacteria was the most negatively affected taxon in response to conversion. This discrepancy in the response of Acidobacteria abundance with our results may be more associated to soil properties of the original forest and land-use type (cropland or pasture) than to the original biome (tropical and subtropical moist broadleaf forest). In fact, pasture systems are known to differ from intensive agriculture and, in that study, converted soils had significantly higher total C and pH than the original forest. Members of Acidobacteria present in the Amazon forest soils are probably specialized to highly acidic environments and, therefore, might be more sensitive to the changes in pH associated with forest conversion. In our study, Salta soils encompass a shorter pH range and forest soils have higher pH values than those from the Amazon [17].

In Jujuy, an area destined to sugarcane crop, the most impacted taxa, and the only one at the phylum level, was Verrucomicrobia. This phylum showed an 80% decrease in abundance in agricultural soils in comparison to forest soils, where it constituted one of the dominant phyla along with Acidobacteria and Proteobacteria. This pattern of low Verrucomicrobia prevalence in agricultural soils and a relatively high abundance in forest soils was also reported by Bergmann et al. [44]. This phylum appears to be dominant in Argentinean subtropical rainforest soils ([45], this study) but not in others rainforests soils (*e*.*g*., Amazon [17, 29] and Borneo [15, 16]). Further studies are needed to find out if these discrepancies in abundance of Verrucomicrobia are explained, at least partially, by soil pH, since Yungas forest soils have higher pH than Amazon and Borneo forest soils. The composition and diversity of bacterial communities are usually influenced by soil properties, like pH, which is known to explain a large proportion of the variance in soil bacterial diversity and community composition at local, regional and continental scales [46, 47].

The ecological relevance of Verrucomicrobia occurrence in our soils is uncertain, as few representatives have been isolated and characterized, and up to now its ecology remains poorly understood. Even though each taxon includes phenotypically, metabolically, and ecologically diverse organisms, so far it is known that some members of this phylum are oligotrophic bacteria, specialized in the degradation of recalcitrant carbon compounds [48]. A recent study comparing bacterial communities in native prairie soils with paired cultivated soils, demonstrated that Verrucomicrobia is a predominant taxa associated with carbon dynamics in the former soils [49]. Given the high abundance of Verrucomicrobia found in Yungas forest soils, and the association of Spartobacteria to these soils according to the IndVal method, we assume they carry out relevant and specialized ecological functions, probably related to C turnover.

Actinomycetales also appeared as indicator OTU for forest soils in Jujuy. However, in rainforest soils from Borneo, this taxon showed an opposite behavior, with increased abundance in oil palm plantations compared to forests [16]. Despite this single result, Actinomycetales are known to be *K*-strategists in soil, specialized in degrading relatively complex, recalcitrant C sources [50], and they were reported to have adapted to environments with low C availability [51]. Therefore, the contrasting responses of Actinomycetales to forest conversion in both studies may be related to the evaluated land-use types.

In long-term agriculture sites from Jujuy, we found four indicator taxa, among which we highlight Betaproteobacteria and Gemmatimonadetes. Despite the ubiquity and prevalence of Gemmatimonadetes in soils, little is known about their physiology or ecology because very few strains have been isolated. Nevertheless, a recent study suggested that members of this phylum are well adapted to drier conditions [52], such as those found in agricultural soils when compared to forest soils. Consistently with our results, Rodrigues et al. [17] also reported an increase in Gemmatimonadetes after land-use change in Amazon forest. Regarding Betaproteobacteria, it is considered to comprise mainly copiotrophic or *r*-strategist forms, with high growth rates and low growth yield efficiency (GYE) [53]. Our previous results comparing physiological profiles of microbial communities from pristine forest and agricultural soils also suggest a predominance of *r*-strategists with low GYE in microbial communities from agricultural soils in the Yungas ecoregion [27]. Such behaviour is expectable because, in most of the cases, agricultural systems are more subjected to regular disturbances. Also, due to the central role that soil microorganisms have on C cycling, microbial communities with low GYE might explain, at least partially, the decrease in soil organic carbon we observed in agricultural soils.

Indicator species analysis also revealed some OTUs associated to different land uses in Salta area. However, as mentioned before, these results should be carefully considered because CAP ordinations did not reveal any association between land use and the structure of soil bacterial communities. Anyhow, we can mention that in forest soils, OTUs from the *Chitinophagaceae* family, ubiquitous chitinolytic microorganisms, appeared as indicator. On the other hand, agricultural soils were dominated by copiotropic Betaproteobacteria (short-term agriculture), as it occurs in long-term agricultural sites in Jujuy, and by OTUs belonging to Nitrospira, Myxococcales and Actinobacteria (long-term agriculture). Again, agricultural soils usually have lower soil moisture compared to forest soils, and this is exacerbated in cropping systems with scarce vegetation cover like soybean crops. In fact, soils from soybean fields in Salta are characterized by Myxobacteria, desiccation-resistant microorganisms that produce stress-resistant myxospores to survive harmful conditions [54].

Diversity of soil bacterial communities was also altered by the conversion of forests to agriculture. When encompassing the whole chronosequence from forest to long-term agriculture, alpha diversity showed an overall increase, as previously reported in other tropical soils [12–15, 17]. In contrast, beta diversity, measured as variation, was significantly reduced in agricultural soils from both areas, especially in soils with long-term agriculture, which bacterial communities showed a more similar composition. Given that our study comprised sites differing in time from deforestation that resemble a chronosequence (*i*.*e*., space-for-time substitution) [55], we are able to draw conclusions on the temporal dynamics of soil microbial communities. For instance, the lower variation in bacterial community structure found in long-term agriculture soils suggests that agriculture leads to their homogenization over time. These findings are consistent with the homogenization of bacterial communities found in a forest-to-pasture conversion from Amazon soils [17]. Conversely, Lee-Cruz et al. [16] reported higher true beta diversity and marginally higher community beta diversity in tropical forest soils of Borneo converted to oil palm plantations. However, Lee-Cruz et al. [16] argue that the observed dissimilarity in bacterial communities within plantation sites may reflect the heterogeneity of the original forest soils before conversion, and that homogenization could occur in the longer term. Moreover, inconsistencies in the response of microbial beta diversity between our results and those observed in Borneo may be related to land-use type. While rainforest soils from the Yungas were cultivated with sugarcane or soybean monocultures, Borneo soils were converted to oil palm plantations. Furthermore, there might be a strong impact of the monoculture management implemented in our soils. Indeed, a recent study from the Argentinean Pampas, investigating the impact of management practices on microbial diversity, proposes that the homogenization of bulk soil bacterial communities is driven by monocultures rather than agricultural activity itself [56]. Although there is uncertainty regarding the impact of rainforest conversion on beta diversity of soil microbial communities, all the reported effects appear to be reliable. This is because, even when this and other studies [16, 17] describe a snapshot in time, beta diversity patterns were reported to be relatively constant over time [57].

The large proportion of OTUs shared between the three land uses (forest, short- and long-term agriculture) in each area, suggests the existence of a core microbiome [58] resistant to disturbances caused by changes in land use and thus, persistent along the chronosequence. Similar findings were reported in soils from the Brazilian Pampas under different land uses [59]. However, in their study, microbial activity was strongly affected in soybean-cropped soils, which exhibited microbial communities with lower metabolic efficiency (higher qCO_2_). In agreement, we detected higher qCO_2_ in soybean soils compared to forest soils [60] that can be related to the dominance of Betaproteobacteria mentioned above. Similarly, Fierer et al. [49], working in prairie soils, argued that communities with many taxa in common also shared many functional attributes. This highlights the importance of linking changes in the structure of soil microbial communities with microbial functionality, as these changes are not always associated.

Soil abiotic properties are known to be intimately associated with bacterial diversity and community composition [15, 47, 61, 62]. However, in our study, while land use had a significant impact in soil organic carbon and total nitrogen, the only chemical variable to be associated with the structure of bacterial communities was soil pH. According to the IndVal method, OTUs belonging to Acidobacteria subgroups 6 and 7 and to Gammaproteobacteria were associated to soil pH. These results agree with a previous study revealing a strongly positive correlation of these Acidobacteria subgroups with pH [63].

We mentioned that pH did not have a significant response to land-use change. However, when relating pH and beta diversity, we found a similar pattern of homogenization; in each area, despite the heterogeneity observed among forest soils, both pH and bacterial community structure were very similar between long-term agricultural sites. Besides pH, some other environmental factors, different from the analyzed soil properties, might be driving the response of microbial communities to changes in land use in Yungas soils. Decreasing organic matter has a profound negative effect on microbial biomass and, in fact, both organic and microbial carbon were reduced after agriculture implementation in our soils [27]. However, in terms of community structure, a core microbiome resisted those drastic changes and persisted after more than 30 years under cultivation, despite the observed changes in the relative abundance of OTUs. It is worth remarking that our results reflect a realistic response of bacterial communities to land-use change, since it was assessed in productive fields with common agricultural activities of the Yungas region. Even though further studies are needed to link the observed changes in the structure of soil microbial communities with soil functionality, the core microbiome would allow maintaining at least some soil ecosystem services after forest conversion to croplands.

## Supporting Information

S1 Fig Venn diagrams comparing soil bacterial communities according to operational taxonomic units (OTUs) defined at distance 0.05. (a) Salta and (b) Jujuy.
(TIF)

S2 Fig Rarefaction curves indicating the observed number of operational taxonomic units (OTUs) defined at distance 0.05 in forest and agricultural soils from Salta (a) and Jujuy (b). F: forest, STA: short-term agriculture, LTA: long-term agriculture.
(TIF)

S1 Table Main chemical properties of the analyzed soil samples. ^a^Soil sample designation refers to their geographical origin (J: Jujuy, S: Salta), farm identification (1 to 3) and land use (F: forest, STA: short-term agriculture, LTA: long-term agriculture).^b^Variables that significantly differed between land-use types within each area (Salta and Jujuy). *P*-values for OC and total N were, respectively, 0.0114 and 0.0021 in Salta, and 0.0006 and 0.0002 in Jujuy.
(DOCX)
