# Supplementary figures and images for: Pyrosequencing Reveals Changes in Soil Bacterial Communities after Conversion of Yungas Forests to Agriculture
Source: PLoS One. 2015 Mar 20;10(3):e0119426. doi: 10.1371/journal.pone.0119426 (PMC4368548; doi:10.1371/journal.pone.0119426)

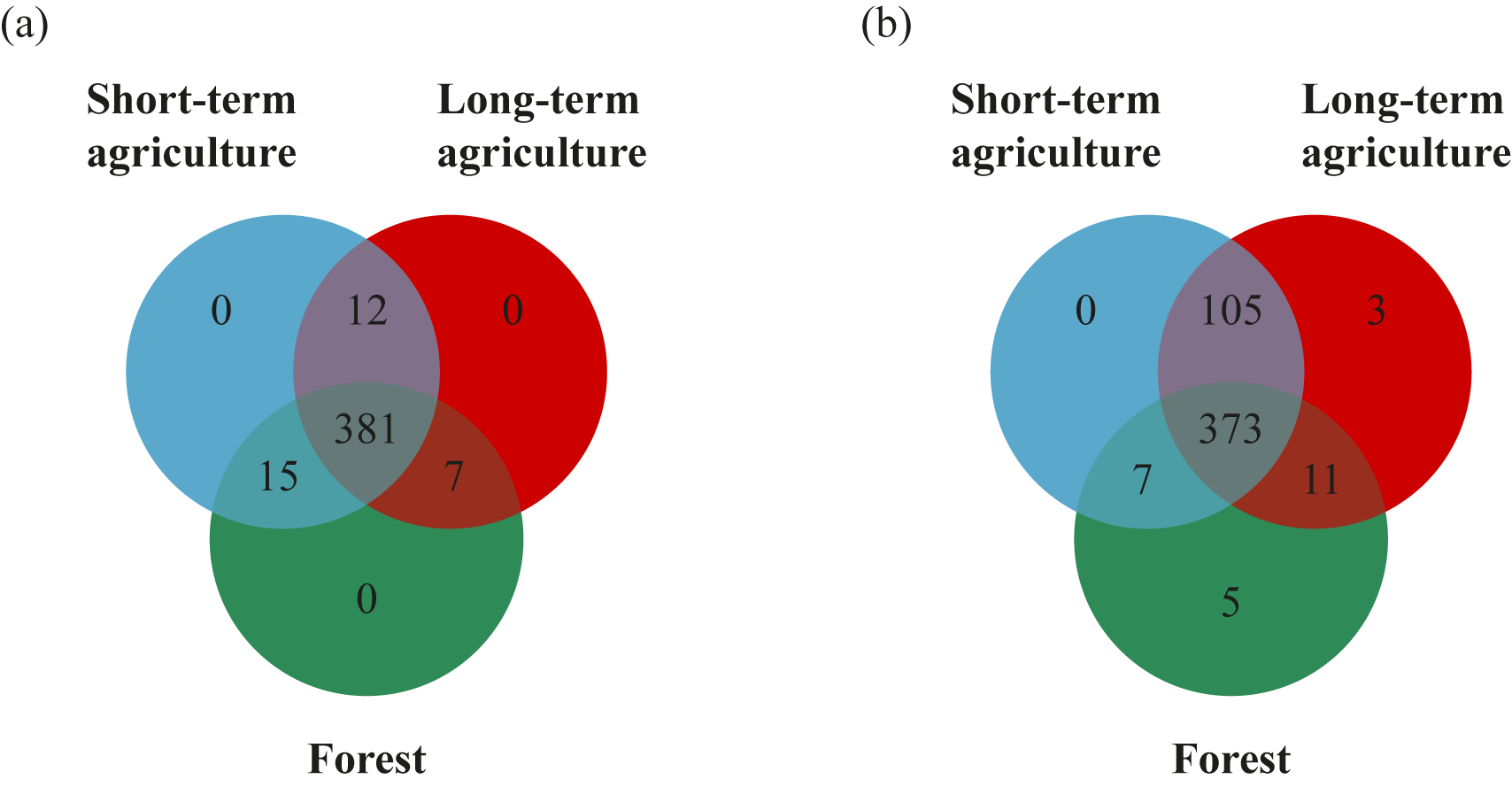

Supplement: S1 Fig — (a) Salta and (b) Jujuy. (TIF) [file pone.0119426.s001.tif]

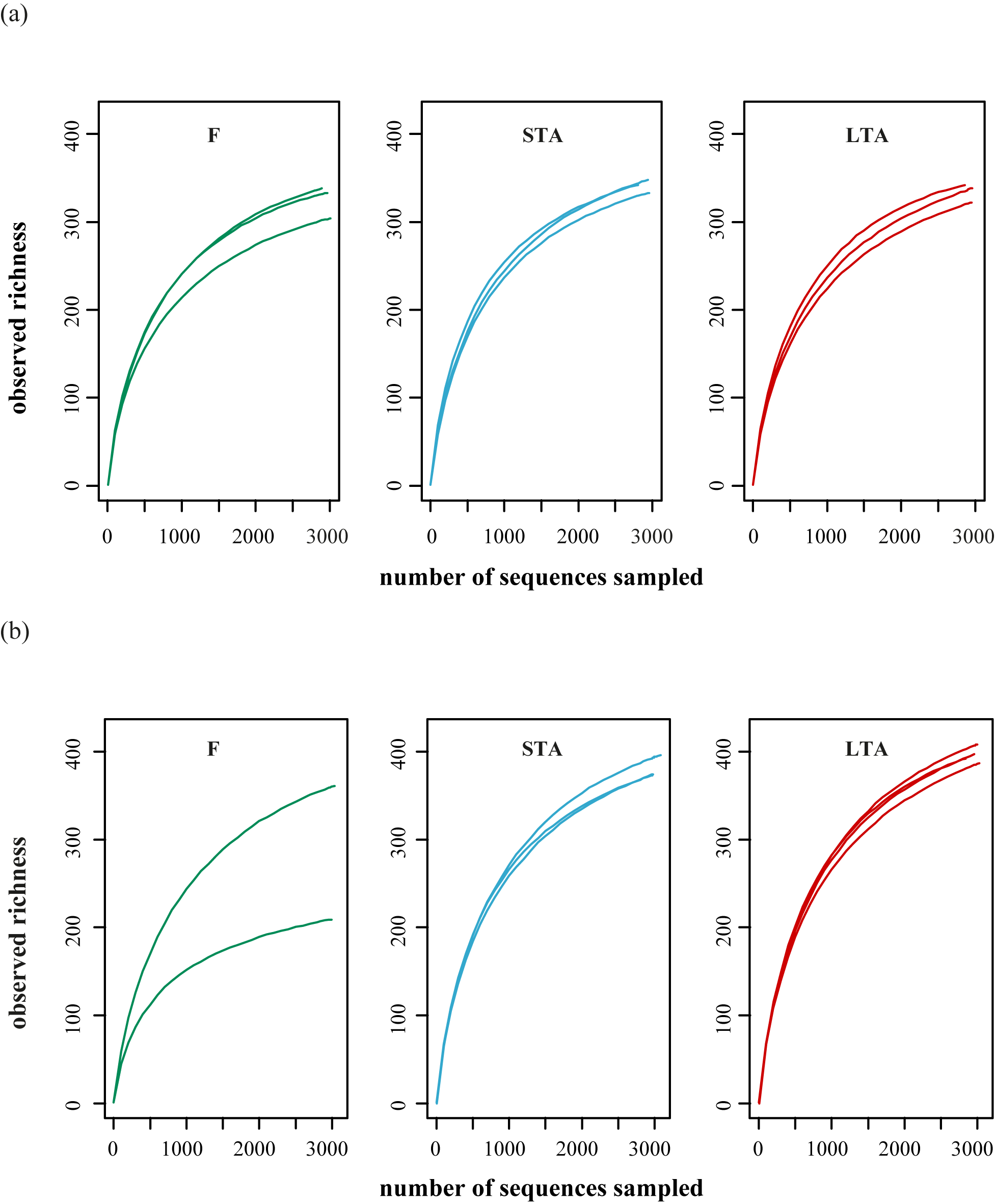

Supplement: S2 Fig — F: forest, STA: short-term agriculture, LTA: long-term agriculture. (TIF) [file pone.0119426.s002.tif]
