# Supplementary material for: Pyrosequencing Reveals Changes in Soil Bacterial Communities after Conversion of Yungas Forests to Agriculture
Source: PLoS One. 2015 Mar 20;10(3):e0119426. doi: 10.1371/journal.pone.0119426 (PMC4368548; doi:10.1371/journal.pone.0119426)
Supplement: S1 Table — aSoil sample designation refers to their geographical origin (J: Jujuy, S: Salta), farm identification (1 to 3) and land use (F: forest, STA: short-term agriculture, LTA: long-term agriculture).bVariables that significantly differed between land-use types within each area (Salta and Jujuy). P-values for OC and total N were, respectively, 0.0114 and 0.0021 in Salta, and 0.0006 and 0.0002 in Jujuy. (DOCX) [file pone.0119426.s003.docx]

| **Sample ID^a^** | **pH** | **Organic C (%)^b^** | **Total N (%)^b^** | **P (ppm)** | **Extractable K (meq 100 g^−1^)** | **Electrical conductivity (mmhos cm^−1^)** |
| --- | --- | --- | --- | --- | --- | --- |
| S1-F | 5.60 | 2.55 | 0.21 | 63 | 1.55 | 0.56 |
| S1-STA | 8.00 | 2.11 | 0.18 | 55 | 1.85 | 0.80 |
| S1-LTA | 6.80 | 1.34 | 0.10 | 29 | 1.22 | 0.36 |
| S2-F | 6.50 | 2.75 | 0.21 | 4 | 0.66 | 0.16 |
| S2-STA | 6.50 | 1.44 | 0.13 | 4 | 0.84 | 0.36 |
| S2-LTA | 6.90 | 1.47 | 0.11 | 50 | 1.44 | 0.28 |
| S3-F | 7.50 | 2.03 | 0.18 | 45 | 1.07 | 0.92 |
| S3-STA | 6.20 | 1.73 | 0.17 | 16 | 0.91 | 0.28 |
| S3-LTA | 6.60 | 1.27 | 0.10 | 8 | 0.82 | 0.16 |
| J1-F | 6.00 | 1.52 | 0.15 | 15.7 | 0.44 | 0.46 |
| J1-STA | 7.62 | 1.05 | 0.12 | 66.4 | 0.87 | 0.55 |
| J1-LTA | 8.12 | 1.01 | 0.08 | 14.7 | 0.49 | 0.94 |
| J2-F | 6.89 | 1.88 | 0.15 | 64.4 | 1.02 | 0.72 |
| J2-STA | 7.87 | 1.12 | 0.12 | 31.5 | 0.49 | 0.53 |
| J2-LTA | 8.18 | 0.81 | 0.07 | 19.2 | 0.39 | 0.51 |
| J3-F | 8.13 | 1.70 | 0.16 | 13.3 | 0.68 | 1.44 |
| J3-STA | 8.30 | 1.06 | 0.12 | 12.1 | 0.2 | 0.53 |
| J3-LTA | 8.22 | 1.11 | 0.09 | 11.5 | 0.37 | 0.62 |
| J3-LTA2 | 7.52 | 1.08 | 0.07 | 9.22 | 0.23 | 0.70 |
| J3-LTA07 | 7.40 | 1.00 | 0.10 | 63.4 | 0.50 | 0.40 |

**Table S1. Main chemical properties of the analyzed soil samples.**

^a^Soil sample designation refers to their geographical origin (J: Jujuy, S: Salta), farm identification (1 to 3) and land use (F: forest, STA: short-term agriculture, LTA: long-term agriculture).

^b^Variables that significantly differed between land-use types within each area (Salta and Jujuy). *P*-values for OC and total N were, respectively, 0.0114 and 0.0021 in Salta, and 0.0006 and 0.0002 in Jujuy.
